# A new generation of versatile chromogenic substrates for high-throughput analysis of biomass-degrading enzymes

**DOI:** 10.1186/s13068-015-0250-y

**Published:** 2015-04-23

**Authors:** Stjepan Krešimir Kračun, Julia Schückel, Bjørge Westereng, Lisbeth Garbrecht Thygesen, Rune Nygaard Monrad, Vincent G H Eijsink, William George Tycho Willats

**Affiliations:** Department of Plant and Environmental Sciences, Thorvaldsensvej 40, Frederiksberg, C 1871 Denmark; Department of Chemistry, Biotechnology and Food Science, Norwegian University of Life Sciences, Chr. M. Falsens vei 1., Aas, 1432 Norway; University of Copenhagen, Faculty of Science, Rolighedsvej 23, Frederiksberg, C 1958 Denmark; Novozymes A/S, Krogshoejvej 36, Bagsværd, 2880 Denmark

**Keywords:** Chromogenic substrates, Carbohydrate-active enzymes, High-throughput screening, Biomass degradation, Plant cell walls, Lytic polysaccharide monooxygenases

## Abstract

**Background:**

Enzymes that degrade or modify polysaccharides are widespread in pro- and eukaryotes and have multiple biological roles and biotechnological applications. Recent advances in genome and secretome sequencing, together with associated bioinformatic tools, have enabled large numbers of carbohydrate-acting enzymes to be putatively identified. However, there is a paucity of methods for rapidly screening the biochemical activities of these enzymes, and this is a serious bottleneck in the development of enzyme-reliant bio-refining processes.

**Results:**

We have developed a new generation of multi-coloured chromogenic polysaccharide and protein substrates that can be used in cheap, convenient and high-throughput multiplexed assays. In addition, we have produced substrates of biomass materials in which the complexity of plant cell walls is partially maintained.

**Conclusions:**

We show that these substrates can be used to screen the activities of glycosyl hydrolases, lytic polysaccharide monooxygenases and proteases and provide insight into substrate availability within biomass. We envisage that the assays we have developed will be used primarily for first-level screening of large numbers of putative carbohydrate-acting enzymes, and the assays have the potential to be incorporated into fully or semi-automated robotic enzyme screening systems.

**Electronic supplementary material:**

The online version of this article (doi:10.1186/s13068-015-0250-y) contains supplementary material, which is available to authorized users.

## Background

Polysaccharide-degrading enzymes including glycosyl hydrolases (GHs) and lytic polysaccharide monooxygenases (LPMOs) are abundant in nature and of major biotechnological importance [1-4]. In particular, the effective utilization of lignocellulosic materials for second-generation biofuels and chemical production is heavily reliant on enzymes for plant cell wall deconstruction [5-7]. Advances in genomics, proteomics, and associated bioinformatics have enabled the identification of vast numbers of putative enzymes currently assigned to over 130 families in the carbohydrate-active enzyme (CAZy) database [8]. However, it is estimated that the activity of no more than 20% of the enzymes in CAZy can be reliably predicted with confidence [9-13], and the gulf between identification and characterisation is rapidly widening. There is therefore a pressing need for fast, cheap and facile techniques to empirically screen enzyme activities and preferably methods that can be integrated with semi- or fully automated colony picking and protein expression systems.

Several techniques already exist for assaying GH and LPMO activities, although all have some limitations. Oligosaccharide products can be analysed using high-performance chromatography, and information on product identities obtained by mass spectrometry [14]. This is a powerful and quantitative approach but low throughput and requires highly specialised equipment and personnel. GH activities can also be monitored by measuring the generation of reducing ends, for example, using the Nelson-Somogyi [15], the 3,5-dinitrosalicylic acid [16], p-hydroxybenzoic acid hydrazide (PAHBAH) [17] and 3-methyl-2-benzothiazolinone hydrazone (MBTH) [18] methods. However, these reducing end assays also have limited throughput and can be prone to side reactions [19]. Chromogenic substrates are available for screening some enzyme activities. For example, para-nitrophenyl (pNP) glycosides can be useful for rapidly assaying GH activities, although these small artificial compounds are of limited use for the study of high molecular weight and sometimes crystalline substrates such as chitin, cellulose and some arabinoxylans [6,20]. However, an elegant new method for the colorimetric detection of chitinase and cellulase activities was recently described [21]. This technique is based on the fact that the oligomeric products of chitinases and cellulases can be modified by chito-oligosaccharide oxidase (ChitO) and the mutant ChitO ChitO-Q268R, respectively, producing hydrogen peroxide (H_2_O_2_). The amount of H_2_O_2_ released is then monitored using a second enzyme, horseradish peroxidase, together with a peroxidase substrate [21]. Azo-dyed and azurine cross-linked (AZCL) polysaccharides are also very widely used for GH screening in a variety of assay types [22,23]. AZCL substrates are produced by cross-linking polysaccharides to render them insoluble and then dyeing them. When exposed to an enzyme with appropriate activity, small dyed oligosaccharide products are released [24]. Assays can be performed in multi-well (usually 96) plates, and activity is detected by the change in colour of enzyme reaction supernatants which can be measured spectrophotometrically. Alternatively, AZCL substrates can be incorporated into agar plates, and activity can be monitored by the formation of coloured halos as the dyed oligosaccharides diffuse into the gel. AZCL-based assays have found widespread usage because of their speed, ease of use and relatively low cost, but they do have some disadvantages, especially in microplate assays [25]. The substrates are only available in one colour (dark blue), limiting throughput since only one substrate can be tested per reaction. Also, they are powders which must be individually weighed into the wells of multi-well plates and undigested substrate particles can interfere with subsequent measurement of coloured supernatants.

We have developed a new generation of chromogenic polysaccharide hydrogel (CPH) substrates based on chlorotriazine dyes that, when used in conjunction with a 96-well filter plate, form a high-throughput assay system. Each substrate can be produced in one of the four colours, and different coloured substrates can be combined in a single well. The use of dyed insoluble polysaccharide substrates has been known for some time, such as in agar plates [26-28] and use of dyed cellulose and starch [29] derivatives. This type of substrates has been used in agar plates [30,31], and this is the first time that a high-throughput assay such as the one we describe has been developed and established. We show here that this methodology can be applied to a wide variety of polysaccharides and proteins and demonstrate its potential for screening GHs, LPMOs and proteases. Importantly, we have also produced chlorotriazine-dyed biomass samples which provide information about substrate availability within the complex polymer mixtures typically encountered by enzymes in industrial contexts.

## Results and discussion

### Development of novel chromogenic hydrogel substrates

**Figure 1:**
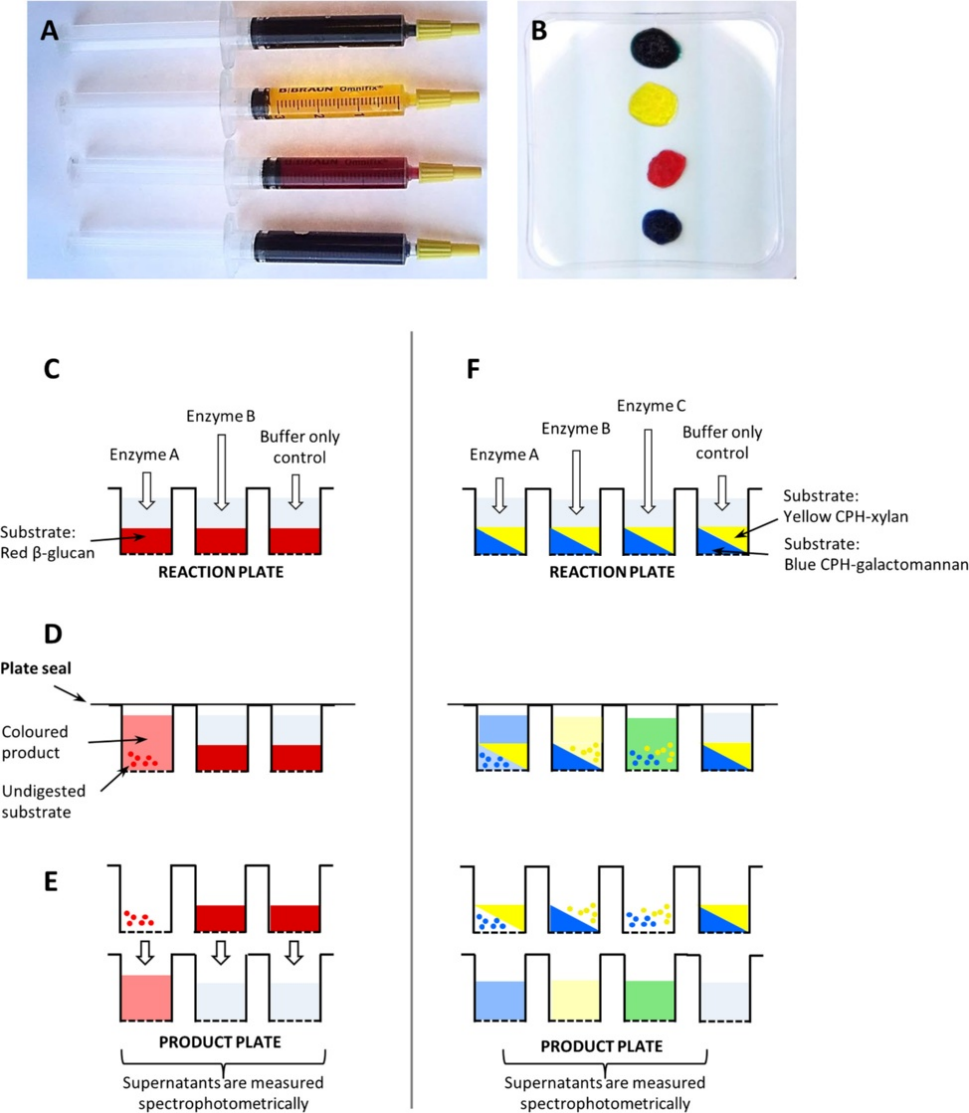
Chromogenic polysaccharide hydrogel (CPH) substrates. **(A)** Examples of CPH substrates loaded into syringe applicators for easy dispensing into microplate wells. (**B)** CPH substrates in four colours, from top green, yellow, red and blue. **(C)** Diagram of part of a reaction plate containing a CPH substrate (red β-glucan) and different enzymes and a control buffer. **(D)** Diagram showing part of a reaction plate during the incubation period. **(E)** Transfer of the supernatant into the product plate prior to spectrophotometric measurement. Result: enzyme A has glucanase activity, and enzyme B does not. **(F)** Diagram showing the simultaneous use of two different coloured CPH substrates (yellow xylan and blue galactomannan). Result: enzyme A has mannanase activity but no xylanase activity, enzyme B has xylanase but no mannanase activity, and enzyme C has both xylanase and mannanase activity.

CPH substrates were produced by first dyeing polysaccharides with one of the four chlorotriazine dyes (red, blue, green or yellow) via nucleophilic aromatic substitution (S_N_Ar) [32]. The polysaccharides were then cross-linked with 1,4-butanediol diglycidyl ether via base-catalysed epoxide opening. The resulting materials are hydrogels which can be easily dispensed using syringes into 96-well filter plates (Figure 1A,B) and be stored for at least 24 months at 4°C without loss of function. A list of the CPH substrates made to date is shown in Table 1, and the enzymes used to test them are detailed in Table 2.

**Table 1:** **List of CPH substrates made and used in the study along with substrate sources**

**Substrate**	**Source**
CPH-2-hydroxyethylcellulose (CPH-HE cellulose)	N/A
CPH-amylopectin	Potato
CPH-amylose	Potato
CPH-arabinan	Sugar beet
CPH-arabinoxylan	Wheat
CPH-casein	Bovine milk
CPH-curdlan	*Alcaligenes faecalis*
CPH-dextran	*Leuconostoc* spp*.*
CPH-galactomannan	Carob
CPH-laminarin	*Laminaria digitata*
CPH-lichenan	Icelandic moss
CPH-pachyman	*Poria cocos*
CPH-pectic galactan	Potato
CPH-pullulan	*Aureobasidium pullulans*
CPH-rhamnogalacturonan I	Potato
CPH-rhamnogalacturonan I (-Gal)^a^	Potato
CPH-rhamnogalacturonan	Soy bean
CPH-xylan	Beechwood
CPH-xyloglucan	Tamarind
CPH-β-glucan from barley	Barley
CPH-β-glucan from oat	Oat
CPH-β-glucan from yeast	Yeast
ICB-Arabidopsis	Rosette leaves from *Arabidopsis thaliana* Col-0 (adult plant)
ICB-bagasse	*Saccharum officinarum* (dried adult plant, stem and leaves)
ICB-fenugreek seeds	*Trigonella* spp*.* seeds
ICB-hemp	*Cannabis* spp*.* (dried adult plant, stem and leaves)
ICB-lupin seeds	*Lupinus angustifolius* seeds
ICB-lupin seeds	*Lupinus angustifolius* seeds
ICB-tobacco	Leaves from *Nicotiana benthamiana* (young plant)
ICB-wheat straw	*Triticum* spp*.* (dried adult plant, stem and leaves)
ICB-willow	*Salix* spp*.* (dried adult plant, milled

^a^(β-1,4-D-galactan side chains removed with endo-galactanase *gal*). N/A, not applicable.

**Table 2:** **Enzymes used in the study**

**Code name**	**Description**	**CAZy family**	**Source**
*ara*	Endo-arabinase (*Aspergillus niger*)	GH43	Megazyme
*cel1*	Endo-cellulase (endo-β-1,4-glucanase) (*Trichoderma longibrachiatum*)	GH7	Megazyme
*cel2*	Cellulase (endo-β-1,4-glucanase) (*Bacillus amyloliquefaciens*)	GH5	Megazyme
*Gal*	Endo-β-1,4-D-galactanase (*Aspergillus niger*)	GH53	Megazyme
*glc*	Endo-β-1,3-glucanase (*Trichoderma* sp.)	GH16	Megazyme
*lic*	Lichenase (endo-β-1,3(4)-glucanase) (*Bacillius* sp.)	GH16	Megazyme
*man*	Endo-β-1,4-mannanase (*Cellvibrio japonicus*)	GH26	Megazyme
*nz1*	Endo-β-1,4-xylanase (*Aspergillus aculeatus*)	GH10	Novozymes
*nz2*	Endo-β-1,4-xylanase (*Thermomyces lanuginosus*)	GH11	Novozymes
*nz3*	Endo-β-1,4-glucanase (*Aspergillus aculeatus*)	GH5	Novozymes
*nz4*	Proprietary fungal endo-β-1,4-mannanase	GH5	Novozymes
*ply1*	Macerase™ Pectinase (*Rhizopus* sp.)	N/A	Calbiochem
*ply2*	Pectolyase Y-23 (*Aspergillus japonicus*)	N/A	Duchefa Biochemie
*ply3*	Pectolyase (*Aspergillus japonicus*)	N/A	Sigma
*pec1*	Pectate lyase (*Cellvibrio japonicus*)	PL10	Megazyme
*pec2*	Pectate lyase (*Aspergillus* sp.)	N/A	Megazyme
*pol1*	Endo-polygalacturonase M2 (*Aspergillus niger*)	GH28	Megazyme
*pol2*	Endo-polygalacturonase M1 (*Aspergillus niger*)	GH28	Megazyme
*rgh*	Rhamnogalacturonan hydrolase (*Aspergillus aculeatus*)	GH28	Novozymes
*xg*	Xyloglucanase (*Paenibacillus* sp.)	GH5	Megazyme
*xyl1*	β-xylanase, M4 (*Aspergillus niger*)	GH11	Megazyme
*xyl2*	Endo-β-1,4-xylanase M1 (*Trichoderma viride*)	GH11	Megazyme
*NcLPMO9C*	Lytic polysaccharide monoxygenase (*Neurospora crassa*)	AA9	N/A
Broth	Culture broth from *Phanerochaete chrysosporium* (3d after inoculation)	N/A	N/A
Proteinase K	Proteinase K (*Tritirachium album*)	N/A	Sigma
Trypsin	Trypsin from bovine pancreas	N/A	Sigma
Elastase	Elastase from porcine pancreas	N/A	Sigma

Code, source and carbohydrate-active enzyme (CAZy) database family. N/A, not applicable.

We developed a rapid assay using the CPH substrates based on 96-well filter plates. Solutions containing enzymes or appropriate buffer controls are added to the wells containing CPH substrates to form the ‘reaction plate’ (Figure 1C). The plate is then sealed and incubated under the desired conditions (Figure 1D). If an enzyme is active against a particular substrate, then soluble, dyed oligosaccharide products are released (some undigested insoluble substrate may remain). The reaction plate is then placed on top of an ordinary 96-well plate (the ‘product plate’), and the two plates are either centrifuged together or placed in a 96-well plate vacuum manifold so that the liquid phase in the reaction plate is transferred through the filter in the bottom to the product plate below (Figure 1E). The absorbance of the products is then measured using a multi-well plate spectrophotometer. The fact that each CPH substrate can be made in four colours may be exploited to increase the throughput of the technique since multiple substrates can be present in a single well. In a theoretical example shown in Figure 1F, a blue CPH-galactomannan and yellow CPH-xylan substrate are combined in each well, so that mannanase and xylanase activities are detected by the release of blue and yellow products, respectively. If both activities are present then a green product is produced. This general approach can be extended so that up to four substrates are combined.

**Figure 2:**
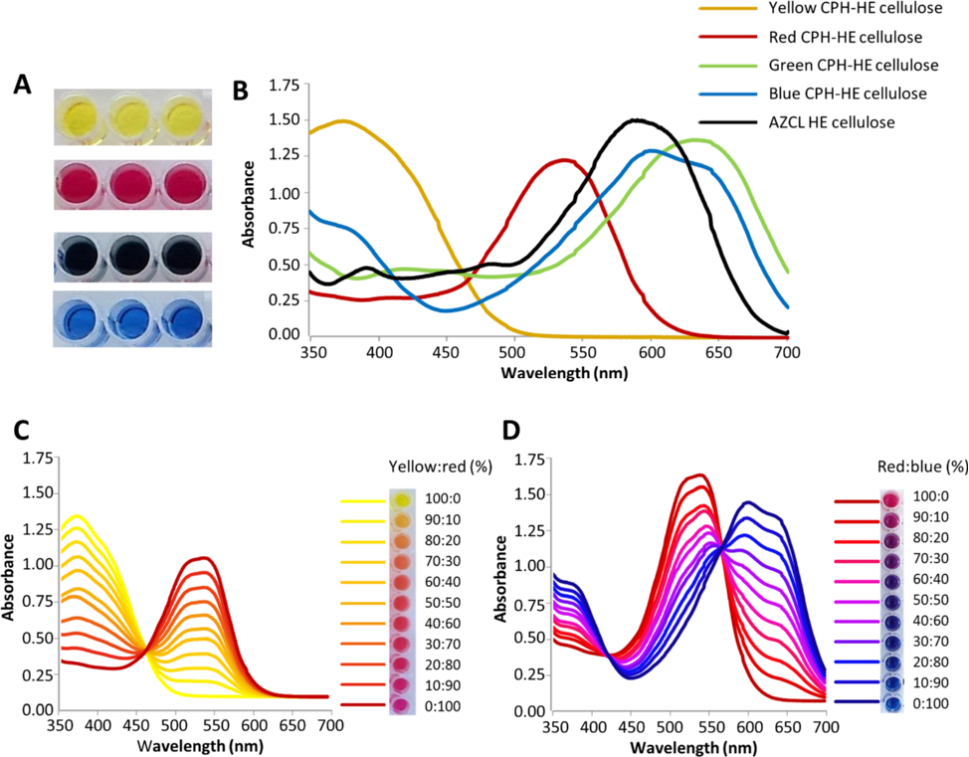
Spectra of the different coloured CPH substrates. **(A)** Product supernatants after a 1-h digestion of four differently coloured versions of CPH-HE cellulose with *endo*-cellulase (*cel1*) used at 2 U/mL in 100 mM sodium acetate buffer, pH 4.5 at room temperature. **(B)** Spectra of product supernatants shown in **(A)**, together with the spectrum for the product of AZCL-hydroxyethyl-cellulose (Megazyme, black line), also digested with the same enzyme at 2 U/mL in 100 mM sodium acetate buffer, pH 4.5 for 1 h at room temperature. **(C)** and **(D)** Spectral response of colour combinations of differently coloured CPH-xylan mixed in a range of 100:0 to 0:100 with 10% increments **(C)** red and blue and **(D)** yellow and blue. See Tables 1 and 2 for details of the substrates and enzymes used.

The soluble products (examples of which are shown in Figure 2A) have distinct spectral properties, and examples of spectral scans of products from red, blue, green and yellow CPH-2-hydroxyethyl-cellulose (CPH-HE cellulose) digested with an endo-cellulase (*cel1*) are shown in Figure 2B. For comparison, the spectrum of products released by digestion of AZCL-HE cellulose with the same enzyme is also shown in Figure 2B. When different coloured substrates are combined in a single reaction with more than one enzyme, then the colour of the products reflects the relative activities of the enzyme mixture against each substrate. Examples of the products of mixed substrates are shown in Figure 2C,D and demonstrate that increments of 10% in colour contribution can be clearly resolved by visual inspection of the products and by differences in the spectra. Linear regression analysis showed that when two substrates with different dyes were present in known ratios in the range from 9:1 to 1:9, this ratio could be estimated within ±5% of the true value based on visible light absorbance spectra of the unmixed coloured supernatants and the spectrum recorded for the mixture. This was done for all two-colour combinations (Additional file 1: Figure S1).

### Stability, reproducibility, detection limits and dose response

**Figure 3:**
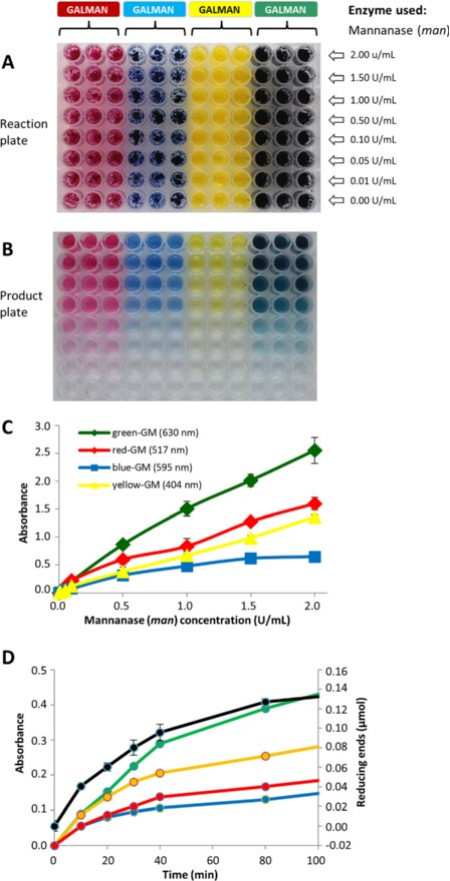
Dose and time responses of CPH substrates. Reaction plate containing four different coloured versions of CPH-galactomannan (GALMAN) - red, blue, yellow and green. **(B)** Product plate containing the products of the digestion of the substrates shown in **(A)** with mannanase (*man*) used in the range of 0 to 2 U/mL (shown to the right of **(A)** in 100 mM potassium phosphate buffer pH 7.0 for 1 h at room temperature. **(C)** Graph showing the absorbance of products released by treatment with mannanase (*man*) at the concentrations shown on the *x*-axis (same conditions as in **(B)** at wavelengths (shown in the legend) appropriate for each product. **(D)** Graph showing the absorbance (*y*-axis to the left) of products released over a time period (from 0 to 100 min) by treatment of four differently coloured versions of CPH-xyloglucan with xyloglucanase (*xg*) at 0.25 U/mL in 100 mM sodium acetate buffer, pH 5.5. Also shown on this graph (*y*-axis to the right) is the production of reducing ends over the same time period from undyed xyloglucan, as measured using the 3-methyl-2-benzothiazolinone hydrazone (MBTH) method. Note that an undyed xyloglucan hydrogel was used since the dye would likely interfere with the MBTH method. See Tables 1 and 2 for details of the substrates and enzyme used.

The reproducibility of the CPH substrate assays was tested by setting up a series of replicate reactions and measuring the variability of the products (Additional file 2: Figure S2). A series of substrates was made in various colours and added in sextuplet to wells in a 96-well reaction plate. Enzymes were added, and after 30-min incubation, the products were collected and quantified at the appropriate wavelength for each colour (Additional file 2: Figure S2A). This procedure was repeated three more times, so that variability both with single plates and across plates was assessed. The quantified products from these experiments (Additional file 2: Figure S2B) are shown in Additional file 2: Figure S2C and demonstrate a high degree of reproducibility (with a standard error of mean (SEM) no greater than 7%). We also tested dose response by varying both enzyme concentration and reaction time (Figure 3). The effect of enzyme concentration is shown for four differently coloured versions of the CPH-galactomannan substrate treated for 1 h with mannanase (*man*) used between 0 and 2 U/mL (Figure 3A,B). The increased colour production can be clearly seen in the product plate (Figure 3B) and quantification of the products revealed that although the absolute absorbance values were different, each substrate yielded an essentially linear response with increasing enzyme concentration (Figure 3C). The release of products over time was assessed by making four different coloured versions of CPH-xyloglucan, digesting with xyloglucanase *xg* at 0.25 U/mL and measuring the absorbances of products formed in 20-min intervals (Figure 3D). In parallel, we also set up the same reaction with undyed xyloglucan and measured the release of reducing ends using the MBTH referred to previously (black line in Figure 3D). Absorbance values were measured at the appropriate wavelengths for each substrate, and again, although the absolute absorbance values varied between the differently coloured versions of the substrate, the trend was the same for each, and this trend closely followed the production of reducing ends from the undyed xyloglucan. The detection limit of the substrates is between 100 and 500 (mU/mL)/h depending on the substrate and the specific activity of the enzymes.

**Figure 4:**
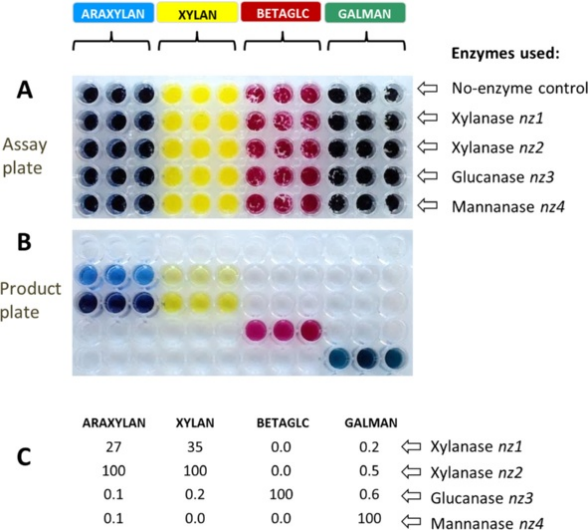
Direct comparison of the output from CPH substrates with the measurement of reducing ends. **(A)** Each well in the reaction plate contained one of the four different CPH substrates: blue CPH-arabinoxylan (ARAXYLAN); yellow CPH-xylan, (XYLAN); red CPH-β-glucan (BETAGLC) and green CPH-galactomannan (GALMAN). The substrates were treated with the enzymes: xylanase *nz1*; xylanase *nz2*; glucanase *nz3* or mannanase (*nz4*) as shown to the right (note that all wells in one row contained the enzymes or control buffer indicted). All enzymes were used at 5 μg/mL in 50 mM sodium acetate buffer, pH 5.5 and incubated at 50°C for 10 min. **(B)** Product plate containing the product of the reaction shown in **(A)**. **(C)** Table showing the relative activities of the enzymes *nz1* to *nz4* as determined by the production of reducing ends when native versions of the polysaccharides shown in **(A)** were treated with enzymes *nz1* to *nz4* used at 5 μg/mL in 50 mM sodium acetate buffer, pH 5.5 at 50°C for 10 min. The highest value was set to 100, and all other values adjusted accordingly. In this case, the PAHBAH reducing end assay was used. See Tables 1 and 2 for details of the substrates and enzyme used.

Most of the enzymes used in the work described here are well-characterized and commercially available (described in Table 2), and data from CPH substrates was in good agreement with previous findings. However, we also validated the technique by making a direct comparison between the performance of the CPH substrates and activity data determined by the PAHBAH method to measure the release of reducing ends [17] (Figure 4). Four purified enzymes were tested: two xylanases, a glucanase and a mannanase (designated *nz1*, *nz2*, *nz3* and *nz4*, respectively) on CPH substrates of blue CPH-arabinoxylan, yellow CPH-xylan, red CPH-β-glucan and green CPH-galactomannan as shown in Figure 4A. Product plate results (Figure 4B) showed that *nz1* and *nz2* had activity against both CPH-arabinoxylan and CPH-xylan with visibly greater colour production for *nz2* against both substrates, and neither enzyme appeared to have activity against CPH-β-glucan or CPH-galactomannan. *nz3* and *nz4* had activity against CPH-β-glucan and CPH-galactomannan, respectively, with no or very little side activity observable. The information about relative activity obtained on the release of reducing ends using the same enzymes with pure (undyed, non-cross-linked) polysaccharides (Figure 4C) was in close agreement with the findings from the CPH substrates with the same major activities detected and very low side activities (Figure 4C).

To verify that the substrates can perform within a wide range of pH values, CPH-HE cellulose and CPH-rhamnogalacturonan were treated with a cellulase (*cel2*) and a pectinase (*pec1*) with activity optima at acidic and basic pH values, respectively. From this experiment, it was apparent that at least for these substrates, the CPH polysaccharides are compatible with a wide range of pH conditions (Additional file 3: Figure S3A). Thermostability of the substrates was tested with CPH-xylan and a thermophilic xylanase (*xyl3*) showing the stability of CPH substrates at high temperatures (Additional file 3: Figure S3B).

We were interested to assess in detail the nature of the products released from CPH substrates. To do this, we used matrix-assisted laser desorption/ionization-time-of-flight-mass spectrometry (MALDI-ToF-MS) to analyse products resulting from the digestion of CPH-xylan (Additional file 4: Figure S4A) and undyed cross-linked xylan (Additional file 4: Figure S4B) with xylanase *xyl1*. The spectrum from the CPH-xylan substrate displayed more near-baseline noise than that of the undyed substrate, and this was presumably due to the presence of the dye molecules. However, in both cases, a range of pentose-containing oligosaccharide products were released that were consistent with xylan fragments. From the mass profiles, we determined that in many cases, xylan oligomers were attached to one or more hydrolysed linker molecules, implying that a significant proportion of the linker was not in fact spanning between polysaccharide chains in either the dyed or undyed version of these substrates (shown schematically in Additional file 4: Figure S4C). Despite this apparent incomplete cross-linking, the substrates were still insoluble and still effective substrates for *xyl1*.

We also tested the storage stability of CPH substrates. A set of reaction plates was made, sealed and stored at 4°C for 1 year. A series of enzyme digestions were made on these plates and compared to identical reactions made in freshly produced reaction plates. As shown in Additional file 5: Figure S5, there was high degree of consistency between the fresh and stored plates.

Taken together, these data indicate that the assays based on CPH substrates are reproducible, stable over time and can tolerate a range of pH and temperature conditions. Like all chromogenic substrates, the CPH polysaccharides are intended for semi-quantitative analysis, but nevertheless, the CPH dose-response profiles show that, for at least the examples tested, the amount of coloured products produced is proportional to the amount of enzyme present and/or incubation times. Moreover, our results from MALDI-ToF analysis suggest that the dying process does not fundamentally alter the nature of the products released.

### Multiplexed assays using mixtures of enzymes and substrates

**Figure 5:**
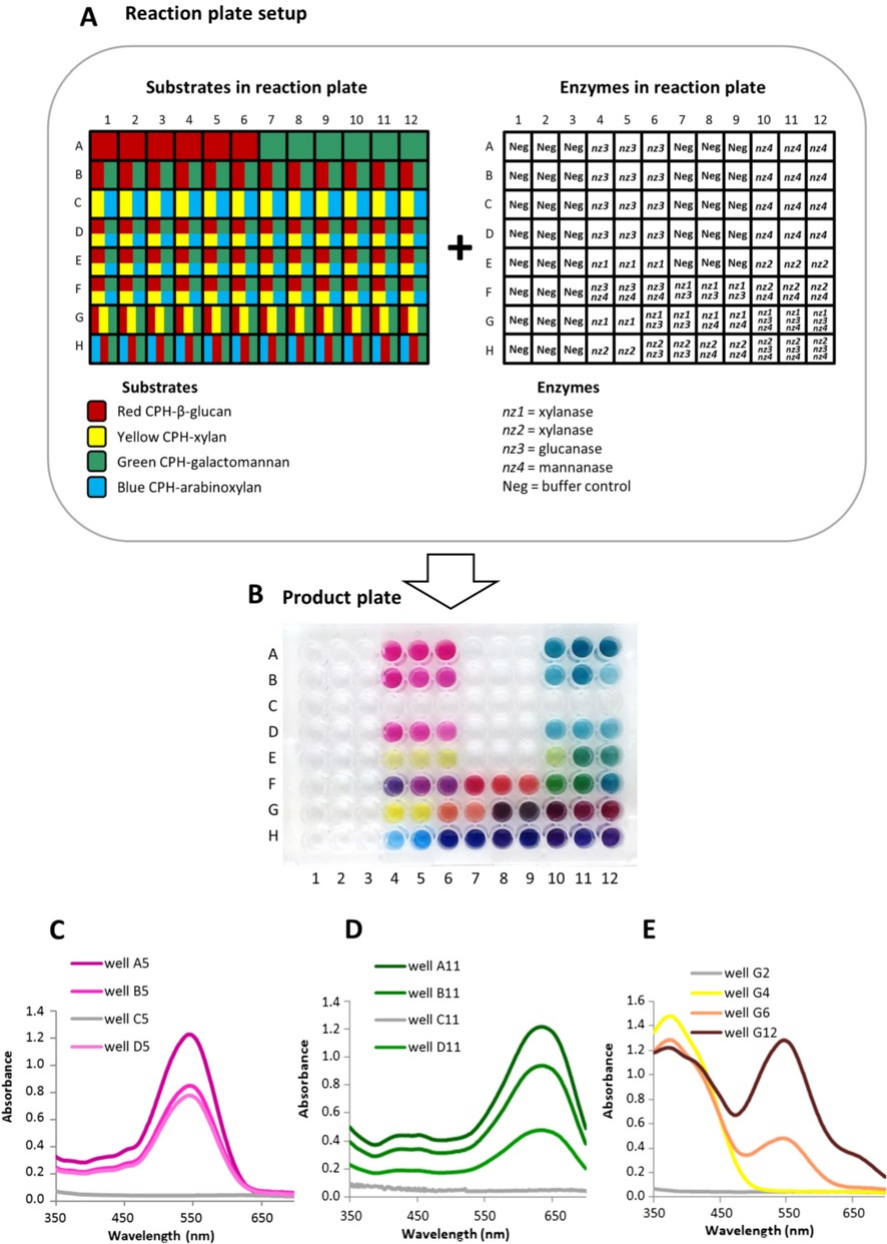
Multiplexed assays using the CPH substrates in different substrate and enzyme concentrations. **(A)** Setup of the reaction plate: CPH substrates used in the experiment were red CPH-β-glucan, green CPH-galactomannan, yellow CPH-xylan and blue CPH-arabinoxylan, and the distribution of these substrates within the reaction plate is shown to the left. Each well contained a single substrate or two, three or four substrates mixed together. Note that the total volume of substrate was the same in each well, and when substrates were mixed in wells, they were present in equal amounts. The distribution of enzymes in the reaction plate is shown to the right, and the enzymes used were xylanase *nz1*, xylanase *nz2*, glucanase *nz3* and mannanase *nz4*. Note that the total amount of enzyme was the same in each well, and when enzymes were mixed in wells, they were present in equal amounts. Some wells contain buffer only (‘Neg’). The reaction buffer used was 50 mM sodium acetate buffer, pH 5.5. **(B)** Image of the product plate containing the products of the reactions shown in **(A)** after a 10-min reaction at 50°C. **(C)** to **(E)** spectra of selected wells as shown in the legends. See Tables 1 and 2 for details of the substrates and enzyme used.

The ability to produce CPH substrates in four different colours is an important feature that can be used to increase the throughput of CPH substrate assays. An example of an assay involving multiple mixed substrates and enzymes is shown in Figure 5. Red CPH-β-glucan, yellow CPH-xylan, green CPH-galactomannan and blue CPH-arabinoxylan were added to wells either alone or mixtures of two, three or four substrates together in the same well (Figure 5A). The *nz* series of enzymes previously characterized (see Figure 4) were added to wells alone or as mixtures of two or three enzymes (Figure 5A). The resulting product plate from these reactions is shown in Figure 5B. Single enzymes acting on single substrates produced the expected corresponding colour of product - but importantly, individual enzyme activities could be resolved by their coloured products even when multiple substrates were present. For example, the product of well A5 had the qualitatively same spectral response as the products in wells B5 and D5, despite the fact that whereas in A5, only red CPH-β-glucan was present, in B5 and D5, the red CPH-β-glucan was mixed with one and three other substrates, respectively (Figure 5C). A similar effect was observed for wells A11, B11 and D11 which produced essentially qualitatively the same spectral responses of a green product, although only well A11 of the substrate plate contained green galactomannan alone (Figure 5D). Note that there are quantitative differences in the absorbance values, and these reflect the fact that when substrates mixed in a single well, then the amount of each individual substrate is proportionally lower since the total volume of substrate material is the same in each well. Thus, the absorbance value for products from well D11 is lower than for well A11 because in D11, the green galactomannan was combined with three other substrates (Figure 5D).

Substrate mixtures also enable multiple enzyme activities to be detected simultaneously. For example, wells G4, G6 and G12 of the substrate plate all contained the same mixture of yellow xylan, green galactomannan and red β-glucan. When xylanase *nz1* alone was added (well G4), then the expected yellow product was produced, but the contribution of both glucanase *nz3* and xylanase *nz1* (well G6) are apparent by the orange product with its distinct spectral response. When mannanase *nz4* was also added (well G12), the contribution of the green product can also be detected by a distinct spectrum (Figure 5E).

### Detecting enzymatic activities in microbial broths

We also tested the utility of the CPH substrates for assessing enzymatic activity in broths of *Penicillium expansum* and *Colletotrichum acutatum* cultured on apple pomace liquid media (Additional file 6: Figure S6). Samples of the crude broths were taken once daily from 1 to 10 days after inoculation. The broths were applied directly to 96-well assay plates containing a variety of CPH substrates including CPH-galactomannan (Additional file 6: Figure S6A), CPH-lichenan, CPH-β-glucan from barley (Additional file 6: Figure S6B and S6C, respectively) and arabinoxylan (Additional file 6: Figure S6D). As expected, broths from both fungi showed a general increase over time in their ability to degrade the substrates, but there were striking differences in the relative activities produced by the two species. After 3 days, there was a sharp increase in mannanase activity in the *C. acutatum* broth (Additional file 6: Figure S6A), whereas the *P. expansum* broth had notably increased activity against lichenan and β-(1,3),(1-4)-glucan from barley (Additional file 6: Figure S6B and S6C). The degradation results for arabinoxylan were similar for both fungi (Additional file 6: Figure S6D).

### CPH substrates can also be used in a simple agar gel system

**Figure 6:**
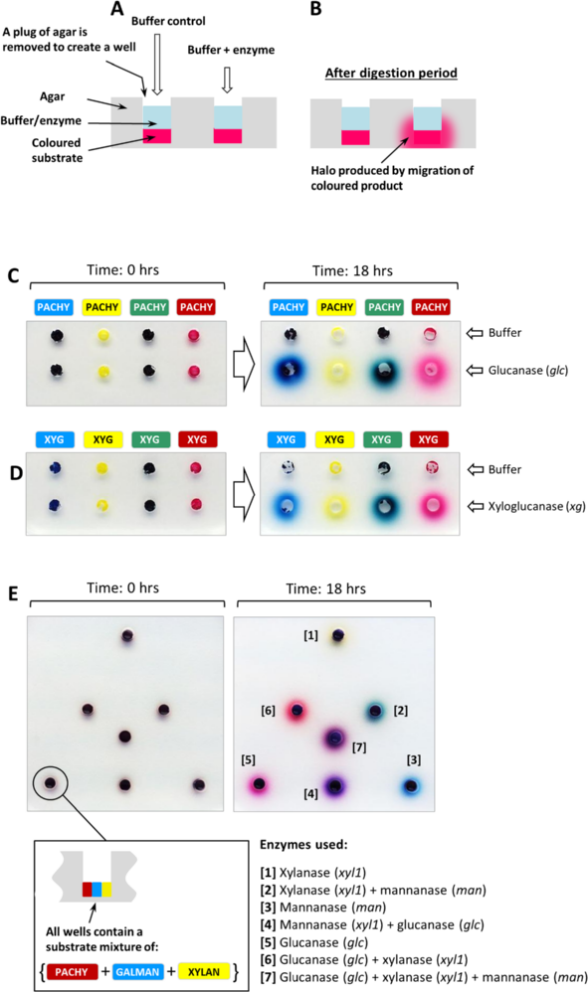
Using CPH substrates in simple agarose plate assays. **(A)** Schematic illustration of the assay using agarose plates. A well is formed in the agarose (either during or after casting), and CPH substrate is added together with an enzyme-containing solution. **(B)** Diagram showing the situation after the digestion period. If the enzyme has activity against the CPH substrate, then a halo is formed by the migration of soluble products. **(C)** and **(D)** examples of agarose plate assays with a glucanase (*glc*) used to digest four differently coloured version of CPH-pachyman (PACHY), and a xyloglucanase (*xg*) used to digest four differently coloured versions of CPH-xyloglucan (XYG), respectively. **(E)** An example of an agar plate assay containing mixed CPH substrates. All the wells contained red CPH-pachyman (PACHY), blue CPH-galactomannan (GALMAN) and yellow CPH-xylan (XYLAN) mixed in approximately equal proportions. Note that different coloured halos are generated depending on what enzyme or combinations of enzymes were present in wells and as indicated in the legend. See Tables 1 and 2 for details of the substrates and enzyme used.

We considered that for some applications, it might be preferable to use a very simple gel-based assay in which enzyme activity is detected by the formation of a coloured halo (Figure 6). This approach is conceptually similar to assays in which particulate AZCL substrates are embedded within agar. Our method differs in that instead of being distributed through the agar, the CPH substrates are contained within a well, either made at the casting stage or subsequently once the agar has set (Figure 6A). If an enzyme has activity against the CPH substrate, then small, soluble products are released that diffuse through the agar and are seen as halos around the wells (Figure 6B). Typically, overnight (18 h) incubations are used, and representative experiments using an *endo*-glucanase (*glc*) and a xyloglucanase (*xg*) are shown in Figure 6C,D. In these experiments, four differently coloured versions of pachyman (Figure 6C) and xyloglucan (Figure 6D) were made and treated with *glc* (see Table 2) and *xg* (see Table 2), respectively. Halos were generated from each substrate version by the respective enzymes, and not by buffer controls.

Using a similar rationale to that described for multi-well plate-based assays, substrates can also be mixed. In the example shown in Figure 6E, all of the wells were filled with the same mixture of three substrates (red CPH-pachyman, blue CPH-galactomannan and yellow CPH-xylan). However, the colours of the halo produced after 18 h depend on the enzyme used, and when more than one enzyme was present, intermediate coloured halos were formed. These experiments showed that although simple, the agar plate-based assays using CPH substrates have the capacity to be highly information-rich.

### Analysis of LPMO and protease activities

**Figure 7:**
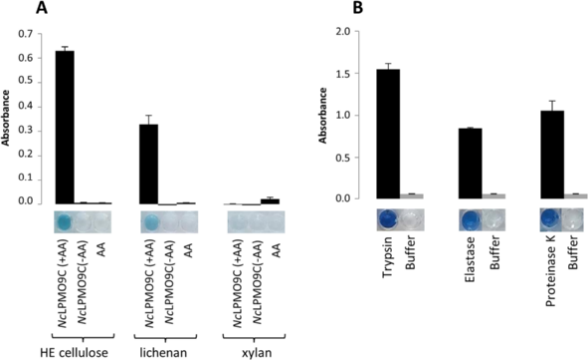
Using CPH substrates to analyse LPMO and protease activities. **(A)** The LPMO *Nc*LPMO9C was tested using three different CPH substrates, from left to right; blue CPH-HE cellulose; blue CPH-lichenan and blue CPH-xylan. *Nc*LPMO9C was tested with and without ascorbic acid (AA, used at 3.2 mM), and ascorbic acid alone was used as a control. Note that activity was observed for the two CPH substrate that contained β-linked glucan (blue CPH-HE cellulose; blue CPH-lichenan) but not blue CPH-xylan. All incubations were in 100 mM potassium phosphate buffer, pH 7.0 incubated for 1 h at 50°C. **(B)** Blue CPH-casein was treated with 0.1 U/mL trypsin, elastase and proteinase K (containing 10 mM Ca^2+^) in 100 mM sodium phosphate pH 7.0 for 20 min at room temperature. See Tables 1 and 2 for details of the substrates and enzyme used.

We further extended the scope of the CPH substrates by assessing if they were compatible with classes of degradative enzyme other than glycosyl hydrolases, specifically an LPMO and different proteases (Figure 7). We used *Nc*LPMO9C, an enzyme that is the first LPMO to be described with activity against both cellulosic and hemicellulosic substrates [4]. Consistent with previous findings, *Nc*LPMO9C released products from CPH substrate versions of CPH-HE cellulose and CPH-lichenan, but not CPH-xylan (Figure 7A). The release of products from CPH-HE cellulose and CPH-lichenan was dependent on the presence of the reducing agent ascorbic acid, since assays lacking ascorbic acid yielded no or negligible products. These data indicate that the activity we observed from *Nc*LPMO9C was indeed mediated by an oxidative reaction. The proteases trypsin, elastase and proteinase K were tested using CPH-casein, and all three enzymes yielded degradation products (Figure 7B).

### Insoluble chromogenic biomass substrates

Enzymes that degrade or modify biomass feedstocks very rarely, if ever, encounter single polysaccharides in isolation. Rather, enzymes usually act upon highly complex mixtures of polysaccharides that are physically intermeshed and sometimes covalently linked. Ideally, assays for screening enzymes intended for biomass deconstruction should reflect this complexity and heterogeneity, and we therefore further extended the concept of chromogenic substrates by producing a series of dyed versions of biomass materials typically used as biofuel/biorefinery feedstocks. Such insoluble chromogenic biomass (ICB) substrates cannot be used to resolve individual enzyme activities since multiple polymers are dyed with the same colour within a single ICB substrate type. However, they can provide information about the ability of an enzyme, cocktail or microbial broth to release oligomeric products from a complex biomass matrix *per se* and as such are a useful addition to the screening toolbox.

ICB substrates are based on raw biomass samples or alcohol-insoluble residue (AIR) preparations which are standard crude preparations of polysaccharides widely used as the starting point for biomass analysis, for example, saccharification assays [33,34]. As a proof of concept, we made six ICB substrates of vegetative material from *Arabidopsis*, tobacco, hemp, willow, wheat straw and bagasse. We also made a further two ICB substrates from fenugreek and lupin seeds (the sources of biomass samples are listed in Table 1). This set of samples was chosen because they represent distinct cell wall types with different polymer compositions and architectures. The *Arabidopsis* and tobacco samples were made from leaf and stem material that is rich in the ‘type I’ primary cell walls found in the younger green parts of the eudicot species. The wheat and bagasse samples predominantly contain the ‘type II’ cell walls typical of commelinid monocots in which β-(1,3),(1,4)-D-glucan and glucuronoarabinoxylans (GAXs) are the prominent hemicelluloses with generally lower levels of pectin. The hemp and willow samples were prepared from older mature organs, which are characterized by secondary cell walls which contain proportionally higher levels of cellulose and xylan than primary cell walls. The lupin and fenugreek seeds are dominated by storage polysaccharides, and lupin seeds are distinctive because β-(1,4)-D-galactan is highly abundant.

The eight ICB substrates were treated separately with a range of enzymes, and the resulting products quantified (Additional file 7: Figure S7). The overall product profiles for *Arabidopsis* and tobacco material were similar, with relatively high signals obtained for pectinolytic enzymes (*ply2* and *ply3*) (Additional file 7: Figures S7A and S7B). Some activity was also observed for xyloglucanase (*xg*) and cellulases (*cel1* and *cel2*), and these data broadly reflect, at least in qualitative terms, the composition of type I primary cell walls. The product profiles from the two commelinid monocot species, wheat and bagasse, were also similar to each other (Additional file 7: Figures S7C and S7D). Compared to *Arabidopsis* and tobacco, much higher signals were obtained by xylanase (*xyl1* and *xyl2*) treatment and this is consistent with the high levels of GAXs in type II cell walls. Interestingly, although the type II cell walls of grasses contain low levels of pectin, higher signals were obtained with the pectolyases *ply2* and *ply3* than for *Arabidopsis* and tobacco. One possible explanation for this is that the signals obtained from ICB substrates are a function of both substrate abundance and accessibility and certain pectins may be less abundant but more accessible in type II cell walls compared to their type I counterparts. Relatively high signals were obtained for hemp and willow (Additional file 7: Figures S7E and S7F respectively) from the xylanases *xyl1* and *xyl2*, and this is consistent with the fact that these species contain type II secondary walls. The unusually high level of β-(1,4)-D-galactan in lupin seeds was readily apparent by comparison of the products produced from the fenugreek and lupin ICB samples (Additional file 7: Figures S7G and S7H, respectively), with abundant product formation by endo-β-(1-4)-galactanase (*gal*) digestion of lupin but not fenugreek ICB samples.

We envisage that, when used in combination, the CPH substrates of defined polysaccharides and ICB substrates can provide insight into what substrate is degraded by an enzyme, to what extent that substrate is available for degradation within a given biomass type. Some caution is required in interpreting data from ICB substrates because we cannot determine to what extent each individual polysaccharide within a biomass sample is dyed, and they are most likely not dyed to an equal extent. Nevertheless, we noted that all the enzymes we used did yield products to some degree, suggesting that most if not all, the polymers were in fact dyed.

We were also interested to analyse what oligomeric products were released from ICB substrates and performed MALDI-ToF-MS analysis of products from hemp and bagasse ICB substrates (Additional file 8: Figure S8A and S8B, respectively) treated with xylanases *xyl1* and *nz2*, respectively. The spectra showed that the enzymes released products similar to those that would be expected for the corresponding natural feedstocks. As expected, xylanases *xyl1* release pentose sugars with or without glucuronic acids depending on the feedstock. Xylans in monocotyledons are typified by arabinosyl- and (4-methyl)-glucuronic acid substituents [35], whereas dicot hardwood xylan contains mainly 4-methyl-glucuronic acid substitutions [36]. Both these types of substitutions limit xylanase activity [37], and consistent with this, we observed longer oligosaccharides which would not be expected in linear xylan devoid of substitutions. Another observation from Additional file 8: Figure S8 is that there are no acetyl-ester modifications present which are common on xylan in these materials [38] and that is due to the high pH used during substrate synthesis which causes hydrolysis of all esters.

## Conclusions

We show here that a new generation of chromogenic substrates have considerable potential for screening single enzymes, enzyme cocktails and microbial broths relevant for biomass deconstruction. Several other chromogenic substrate-based assays have been described, but the CPH and ICB substrates have some distinct features that make them a valuable addition to existing screening technology. Because they are hydrogels rather than powders, the CPH substrates are convenient to handle and quick and easy to distribute into assay plates. The ability to produce both CPH and ICB substrates in several colours supports high-throughput multiplexed assays whereby multiple enzymes or side activities of a single enzyme can be detected in a single reaction well. This means that CPH-based assays have the capacity to be highly information-rich compared to other simple assay systems. Furthermore, to our knowledge, the ICB substrates represent the first attempt to produce a rapid assay system in which the natural multi-polymer complexity of biofuel feedstocks is represented. Our data also showed that both CPH and ICB substrates have potential for LPMO and protease screening.

However, in common with other insoluble chromogenic substrates, the CPH and ICB reagents also have limitations. For example, ester bonds are not preserved, so esterase activities cannot be tested. Also, as with AZCL polysaccharides, the CPH and ICB substrates are not reactive with *exo*-acting enzymes, presumably because of steric hindrance of cleavage sites and alternative methods may need to be employed for assaying that class of GHs. It is also important to recognise that although spectrophotometric analysis of the CPH and ICB products provides quantitative information about relative activity levels, these assays are not intended to replace fully quantitative biochemical techniques to measure absolute activity values. We envisage that the 96-well assays we have developed will be used primarily for first-level screening of large numbers of putative CAZymes, and the assays have the potential to be incorporated into fully or semi-automated robotic enzyme screening systems.

## Methods

### Reagents, enzymes and microorganisms

Amylose, 2-hydroxyethyl-cellulose (Sigma 434965, molar 2-hydroxyethyl substitution 2.5 mol per mol cellulose), curdlan, laminarin, amylopectin, pectolyase from *A. japonicus* and α-amylase from bovine pancreas were obtained from Sigma (Brøndby, Denmark). All other polysaccharides were obtained from Megazyme (Bray, Ireland). The enzymes used are listed with the supplier in Table 2. The dyes reactive red 4, reactive blue 4, reactive green 19 and reactive yellow 2, cross-linker 1,4-butanediol diglycidyl ether, NaOH and all salts for buffers were obtained from Sigma (Brøndby, Denmark). Two pathogenic fungi *C. acutatum* (isolate SA 0-1) and *P. expansum* (isolate IK2020) and the apple pomace media were kindly provided by Birgit Jensen and Daniel Buchvaldt Amby (Department of Plant and Environmental Sciences, Faculty of Science, University of Copenhagen, 1871 Frederiksberg, Denmark).

### Production of CPH and ICB substrates

Synthesis of CPH substrates was performed according to a protocol modified from Ten *et al*. [30]. The polymer was mixed with 0.5 M NaOH (concentrations from 3% to 20% *w*/*V*), and the sample was dissolved by shaking (110 rpm) at 60°C (or room temperature). Samples were then dyed by adding 0.5 g of chlorotriazine dye followed by incubation at 60°C (or at room temperature for substrates soluble at room temperature) for 0.5 to 4 h with shaking. Cross-linking of the dyed polysaccharide solutions was achieved, after being cooled down to room temperature if needed, by adding 1,4-butanediol-diglycidyl ether. The cross-linker concentration ranged from 1.2% to 16% (*V*/*V*) and was optimized to provide maximum hydrogel responsiveness for enzyme digestion whilst preserving optimal physical consistency for handling and the optimal amount it varied greatly from polysaccharide to polysaccharide. The reaction mixture was vortexed vigorously for 2 min and then left to stand for 48 to 96 h at room temperature without agitation for hydrogel formation. To enable more efficient purification and handling, the resulting hydrogels were homogenized within the tube using a spatula to a paste-like material and the material was transferred onto a nylon membrane mesh (31-μm mesh) placed over a Büchner funnel where it was washed with boiling sterile water until no more free dye was released, left to stand until there was no more water draining from the funnel, collected into a fresh tube, and stored at 4°C. In the case of the casein protein substrate, the last wash was with 0.01% NaN_3_ in sterile water. Production of ICB substrates was based on the same dyeing chemistry as for the CPH substrates, but the cross-linking step was omitted. Briefly, 2 g of plant material (freeze-dried and crushed raw and not pre-treated) and 500 mg of dye were mixed and suspended in 10 to 20 mL of 0.5 M NaOH (the volume was adjusted so that the suspension was free-flowing). The reaction mixture was shaken for 4 h at room temperature. After cooling to room temperature, the substrates were cleaned similarly as for the CPH substrates above. After an isopropanol wash, samples were stored suspended in isopropanol at 4°C.

Effects of dye concentration have not been studied in detail, especially because dyes used were of technical grade, but the general observation was that there is no observable change in substrate properties depending upon dye concentration within the concentration range tested (5% to 20%, *w*/*V*).

### Enzyme assay in 96-well filter plates

Enzyme activity assays in 96-well plate format were performed by transferring 100 μL CPH or 1 mg ICB substrates into a 96-well ‘reaction’ plate (filter-plate MSHVN4510, Millipore, VWR, Herlev, Denmark) where they were washed once with water. Buffer (100 mM, sodium acetate or potassium phosphate buffer, pH dependent on the individual enzyme) containing enzyme (0.5 to 2 U/mL) was added to each well to a final volume of 150 μL. The plate was sealed using adhesive PCR plate seals (Thermo Scientific, VWR, Herlev, Denmark) and incubated for 10 min to 1 h at room temperature with shaking. The reaction plate was then place on top of another 96-well plate (the ‘product plate’, MSCPNUV40, Millipore, Herlev, Denmark), and supernatants containing products were transferred by centrifugation at 2,700 *g* for 10 min or by using a vacuum manifold. Absorbances were measured at the *λ*_max_ for each substrate colour (404 nm for yellow, 517 nm for red, 595 nm for blue and 630 nm for green) using a plate reader (SpectraMax M5, Molecular Devices, Sunnyvale, USA).

### CPH assays in agar plates

Agar plates were cast containing 23 mM Britton-Robinson buffer (pH 6.0), 1% agar and 0.01% NaN_3_. Holes were made in the agar with a metal cylinder borer (diameter 6 mm). CPH substrates (prepared as described above) were diluted (300 mg CPH substrate with 500 μL water) and homogenized using a sample disruptor (TissueLyser II, Qiagen AB, Sollentuna, Sweden) at 30 Hz for 15 min to form a fluid-like gel suspension. Sixty microliters of each substrate was transferred to each hole. The wells were topped with 0.75% agar. To perform the assay, 20 μL 10 U/mL of enzyme in appropriate buffer was added and the plate was incubated overnight at 30°C, after which halos were examined.

### Analysis of culture broth from fungi

Two pathogenic fungi *C. acutatum* (isolate SA 0-1) and *P. expansum* (isolate IK2020) were cultivated following the protocol described in Vidal-Melgosa *et al.* 2015 [39]. Supernatants were collected every day, frozen in liquid nitrogen and stored at −80°C until use. To assay enzyme activities in the broths, 5 μL of broth was added to 100 mM sodium acetate buffer pH 4.5 to a final volume of 155 μL. This solution was then used to perform CPH substrate assays as describe above with an incubation time of 1 h and at room temperature. Four independent replicates of each sample were performed.

### Mass spectrometry

For mass spectrometry analysis of products, 2 μL of a 9 mg/mL mixture of 2,5-dihydroxybenzoic acid (DHB) in 30% acetonitrile was applied to a MTP 384 ground steel target plate TF (Bruker Daltonics GmbH, Bremen, Germany). One-microliter sample was then mixed into the DHB droplet and dried under a stream of air. The samples were analysed with an Ultraflex2 MALDI-ToF/ToF instrument (Bruker Daltonics GmbH, Bremen, Germany) equipped with a Nitrogen 337-nm laser beam. The instrument was operated in positive acquisition mode and controlled by the FlexControl 3.3 software package. All spectra were obtained using the reflectron mode with an acceleration voltage of 25 kV, a reflector voltage of 26, and pulsed ion extraction of 40 ns in the positive ion mode. The acquisition range used was from *m/z* 300 to 3,000. The data was collected from averaging 400 laser shots, with the lowest laser energy necessary to obtain sufficient signal-to-noise ratios. Peak lists were generated from the MS spectra using Bruker FlexAnalysis software (Bruker Daltonics GmbH, Bremen, Germany) (Version 3.3).

## Additional files

Additional file 1: Figure S1. Linear regression analysis of 2-colour combinations of CPH substrates*.* The graphs show linear regression analysis from spectra of two-colour combinations of degradation products (the supernatant) from CPH-xylan mixed in ratios from 0% to 100% for each. The deviation of the data points from the line shows deviation from linearity. The results of this analysis show that the true ratio can be determined using linear regression within a ±5% error margin. Additional file 2: Figure S2. Reproducibility of the high-throughput assay using CPH substrates*.* (A) Four identical reaction plates were set up as shown. The substrates in the reaction plate are shown to the left, and the enzymes in the reaction plate are shown to the right. The substrates used were red CPH-arabinan, green CPH-xylan, yellow CPH-HE cellulose, blue CPH-β-glucan, red CPH-pectic galactan, green CPH-lichenan, red CPH-xyloglucan and yellow CPH-galactomannan. The enzymes used were arabinanase (*ara*), xylanase (*xyl1*), glucanase (*glc*), galactanase (*gal*), lichenanase (*lic*) xyloglucanase (*xg*) and mannanase (*man*). (B) The four product plates containing the products from the four separate reaction plates. (C) Graph showing the mean absorbances from the product plates (measured at *λ*_max_ for each substrate colour). See Tables 1 and 2 for details of the substrates and enzyme used. The reaction was performed for 30 min at room temperature in 100 mM sodium acetate buffer pH 4.5 for *ara, xyl1, glc and gal*, in 100 mM sodium phosphate buffer pH 7.0 for *lic* and *man* and in 100 mM sodium acetate buffer pH 5.5 for *xg*. Additional file 3: Figure S3. pH and temperature stability of CPH substrates. (A) Graph showing pH responses for green CPH-HE cellulose and green CPH-rhamnogalacturonan treated with a cellulase (*cel2*) and a pectinase (*pec1*), respectively, to show the usage of the CPH substrates at a pH range between pH 3.0 to 10.0. The assay was performed with 1 U/mL enzyme concentration in 100 mM Britton-Robinson buffer at room temperature for 30 min. (B) Graph showing a temperature stability test. Green CPH-xylan was treated with a thermophilic xylanase (*xyl3*, 0.1 U/mL) in sodium acetate buffer pH 6.0 for 1 h from 20°C to 80°C. See Tables 1 and 2 for details of the substrates and enzyme used. Additional file 4: Figure S4. Mass spectra of products from CPH-xylan digestion. (A) Mass spectrum of dyed CPH-xylan digested by xylanase *xyl1* where the oligomers are composed of *x* pentose units and *y* hydrolysed linker units (Pent*x*linker*y*). (B) Mass spectrum of undyed cross-linked xylan digested by xylanase *xyl1* where the oligomers are composed of *x* pentose units and *y* hydrolysed linker units (Pent_*x*_linker_*y*_). (C) Theoretical scheme showing the possible origins of the products observed in (A) and (B). Additional file 5: Figure S5. Storage stability of CPH substrates. The storage stability of CPH substrates was tested by comparing the output from fresh and year-old reaction plates. (A) Images showing products from fresh and year-old reaction plates in which each well contained a mixture of the substrates shown to the right. The enzymes used are indicated at the top. (B) Another second example of an experiment comparing the output from fresh and year-old plates. Each well contained a mixture of the substrates shown to the right. The enzymes used are indicated at the top. See Tables 1 and 2 for details of the substrates and enzyme used. Additional file 6: Figure S6. Using CPH substrates to analyse enzyme activity in crude fungal broths. The two pathogenic fungi *Penicillium expansum* and *Colletotrichum acutatum* were cultivated over 10 days, and enzyme activities in the culture broth were analysed using four different CPH substrates: (A) yellow CPH-galactomannan, **(**B) yellow CPH-lichenan, (C) yellow CPH-β-glucan from barley, (D) yellow CPH-arabinoxylan. As a negative control, the substrates were also treated with broth alone (grey lines). The reaction was incubated in 100 mM sodium acetate buffer pH 4.5 for 1 h at room temperature. See Table 2 for details of the substrates used. Additional file 7: Figure S7. Insoluble chromogenic biomass substrates. A range of insoluble chromogenic biomass substrates were produced (all using a red-coloured dye) and treated with a range of enzymes (shown on *x*-axes). The absorbance values are means from three wells measured at 517 nm. Enzymes were used at 10 U/mL for 24 h at room temperature. The assay was performed using 100 mM sodium acetate buffer pH 4.5, except for the enzymes *ply1*, *ply2*, *ply3*, *pol1* and *xg* used at pH 5.5, for *cel2* pH 6.0, for *pec3* 100 mM sodium phosphate pH 7.0 and for *pec1* sodium carbonate pH 10.0. The substrates used were (A) tobacco, (B) *Arabidopsis*, (C) wheat straw, (D) bagasse, (E) hemp, (F) willow, (G) fenugreek seeds and (H) lupin seeds. See Table 2 for details of the enzyme used. Additional file 8: Figure S8. MALDI-ToF MS spectra of released products during enzyme treatments of insoluble chromogenic biomass substrates. (A) *MALDI-ToF MS spectra* of the products released from hemp insoluble chromogenic biomass substrates treated with xylanase *xyl1*. **(**B) *MALDI-ToF MS spectra* of the products released from bagasse treated with xylanase *nz2*. MeGlcA_*x*_Pent_*y*_ represent oligosaccharides with *x* number of 4-methyl-glucuronic acid and *y* number of xylose residues; Pent_*x*_ = pentose oligosaccharides (most likely (arabino-)xylooligosaccharides, arabinosylations limiting complete degradation by the endoxylanases *xyl1* and *nz2*) containing *x* number of pentoses; asterisk (*) represents sodium adducts, which commonly occur for uronic acids.

## Abbreviations

AZCL: azurine cross-linked
CPH: chromogenic polysaccharide hydrogel
GAX: glucuronoarabinoxylan
GH: glycosyl hydrolase
ICB: insoluble chromogenic biomass
LPMO: lytic polysaccharide monooxygenase
MALDI-ToF-MS: matrix-assisted laser desorption/ionization-time-of-flight-mass spectrometry
SEM: standard error of mean
