# Supplementary figures and images for: A new generation of versatile chromogenic substrates for high-throughput analysis of biomass-degrading enzymes
Source: Biotechnol Biofuels. 2015 Apr 23;8:70. doi: 10.1186/s13068-015-0250-y (PMC4428106; doi:10.1186/s13068-015-0250-y)

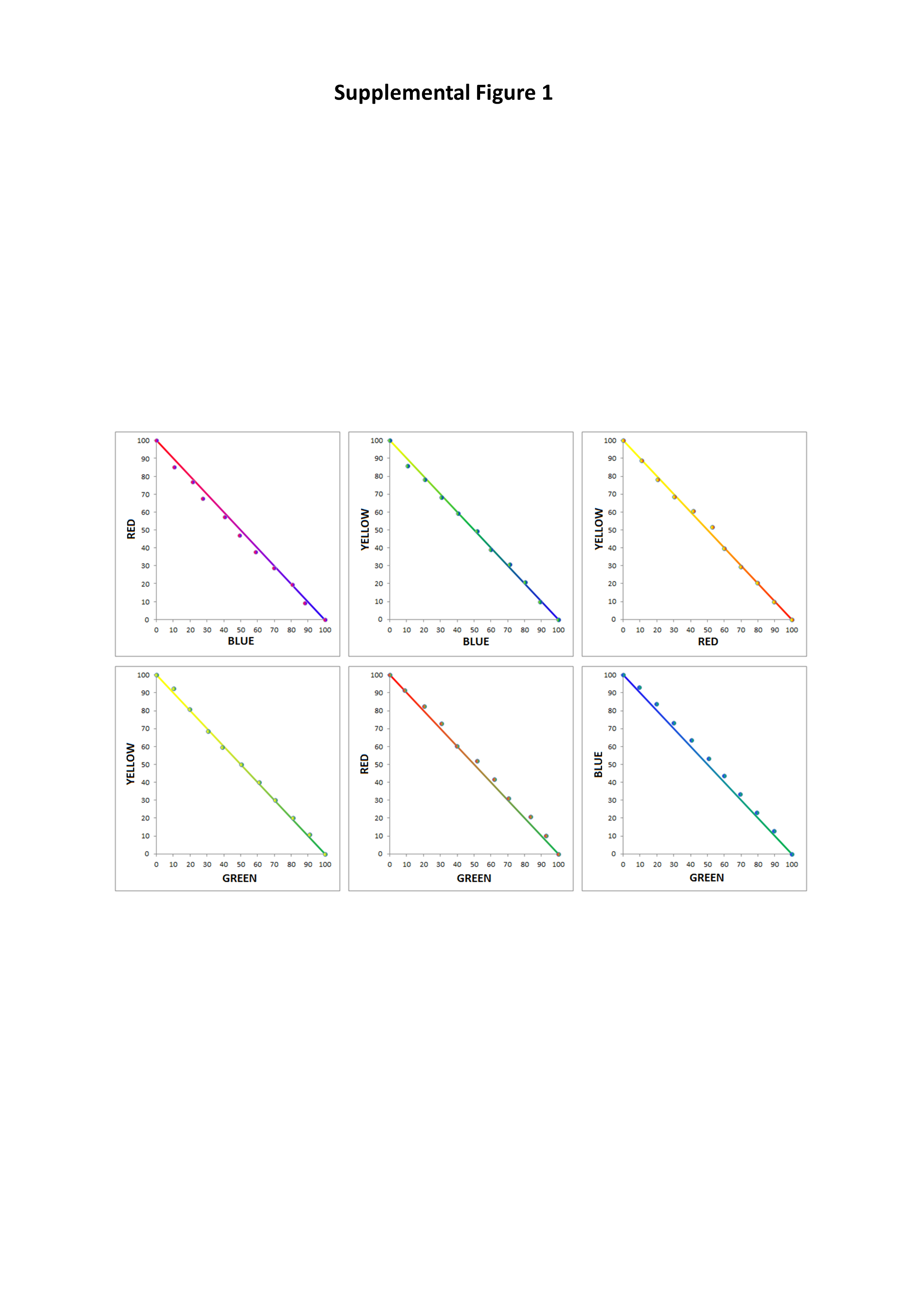

Supplement: Additional file 1: Figure S1. — Linear regression analysis of 2-colour combinations of CPH substrates. The graphs show linear regression analysis from spectra of two-colour combinations of degradation products (the supernatant) from CPH-xylan mixed in ratios from 0% to 100% for each. The deviation of the data points from the line shows deviation from linearity. The results of this analysis show that the true ratio can be determined using linear regression within a ±5% error margin. [file 13068_2015_250_MOESM1_ESM.tif]

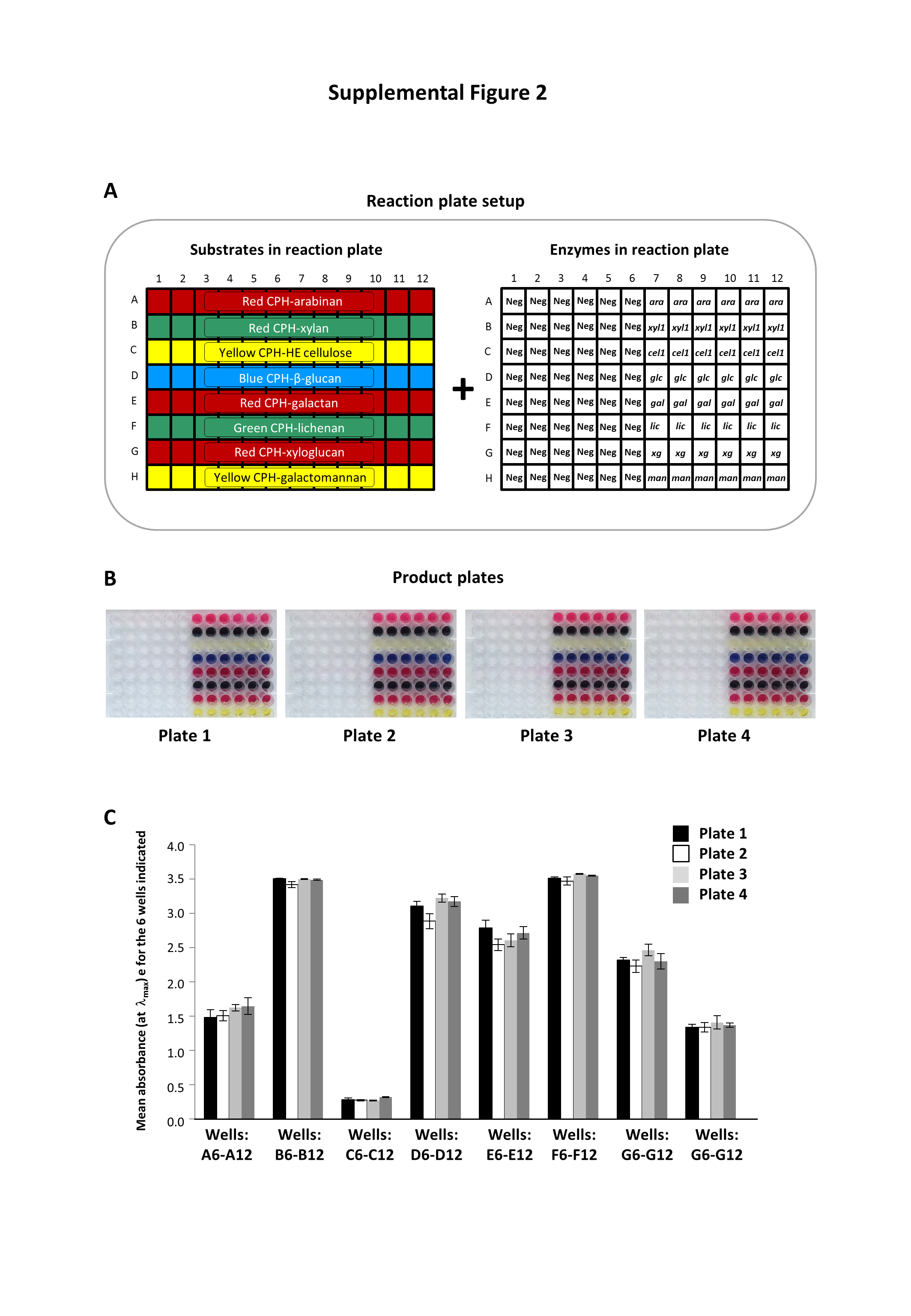

Supplement: Additional file 2: Figure S2. — Reproducibility of the high-throughput assay using CPH substrates. (A) Four identical reaction plates were set up as shown. The substrates in the reaction plate are shown to the left, and the enzymes in the reaction plate are shown to the right. The substrates used were red CPH-arabinan, green CPH-xylan, yellow CPH-HE cellulose, blue CPH-β-glucan, red CPH-pectic galactan, green CPH-lichenan, red CPH-xyloglucan and yellow CPH-galactomannan. The enzymes used were arabinanase (ara), xylanase (xyl1), glucanase (glc), galactanase (gal), lichenanase (lic) xyloglucanase (xg) and mannanase (man). (B) The four product plates containing the products from the four separate reaction plates. (C) Graph showing the mean absorbances from the product plates (measured at λ max for each substrate colour). See Tables 1 and 2 for details of the substrates and enzyme used. The reaction was performed for 30 min at room temperature in 100 mM sodium acetate buffer pH 4.5 for ara, xyl1, glc and gal, in 100 mM sodium phosphate buffer pH 7.0 for lic and man and in 100 mM sodium acetate buffer pH 5.5 for xg. [file 13068_2015_250_MOESM2_ESM.tif]

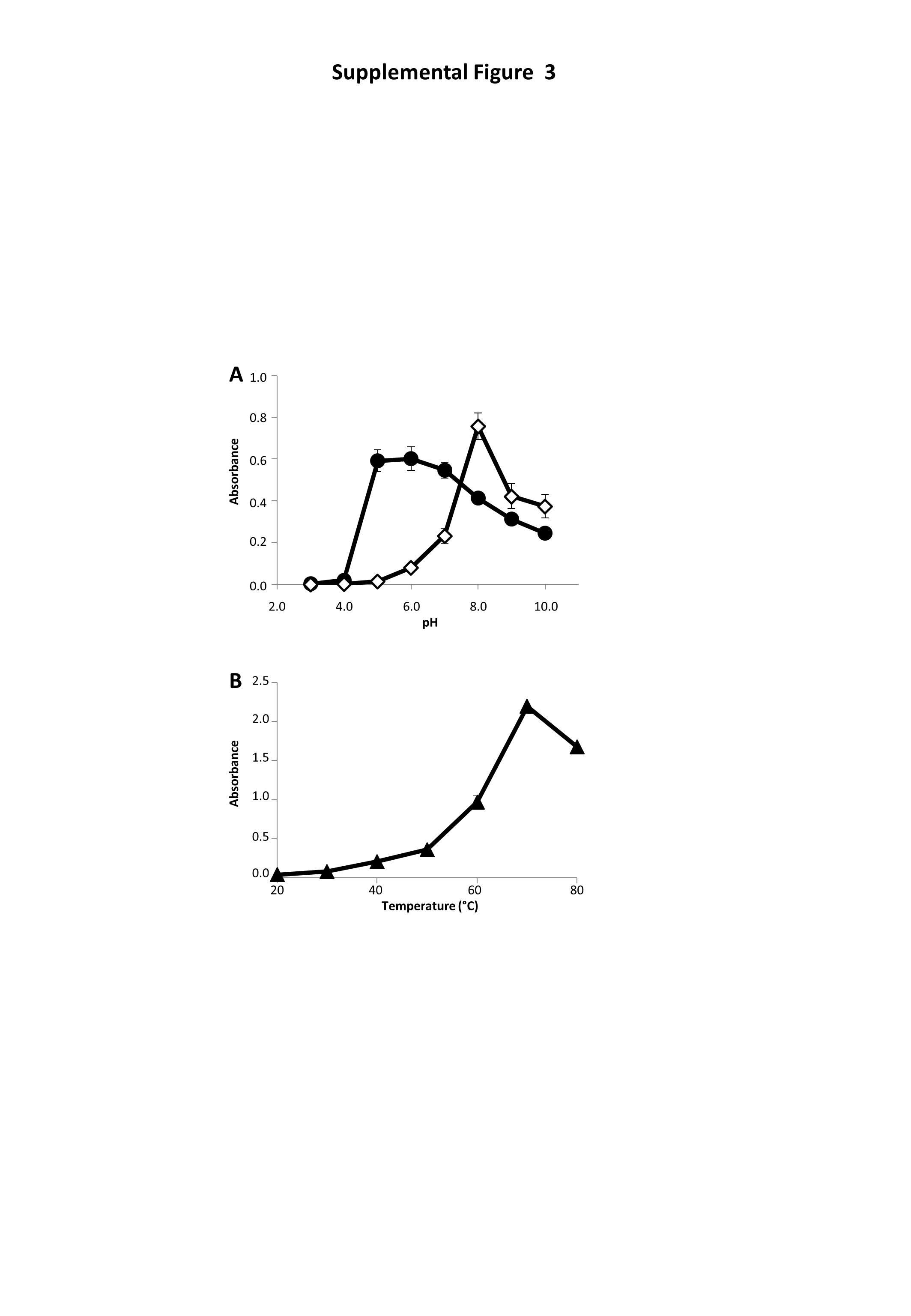

Supplement: Additional file 3: Figure S3. — pH and temperature stability of CPH substrates. (A) Graph showing pH responses for green CPH-HE cellulose and green CPH-rhamnogalacturonan treated with a cellulase (cel2) and a pectinase (pec1), respectively, to show the usage of the CPH substrates at a pH range between pH 3.0 to 10.0. The assay was performed with 1 U/mL enzyme concentration in 100 mM Britton-Robinson buffer at room temperature for 30 min. (B) Graph showing a temperature stability test. Green CPH-xylan was treated with a thermophilic xylanase (xyl3, 0.1 U/mL) in sodium acetate buffer pH 6.0 for 1 h from 20°C to 80°C. See Tables 1 and 2 for details of the substrates and enzyme used. [file 13068_2015_250_MOESM3_ESM.tif]

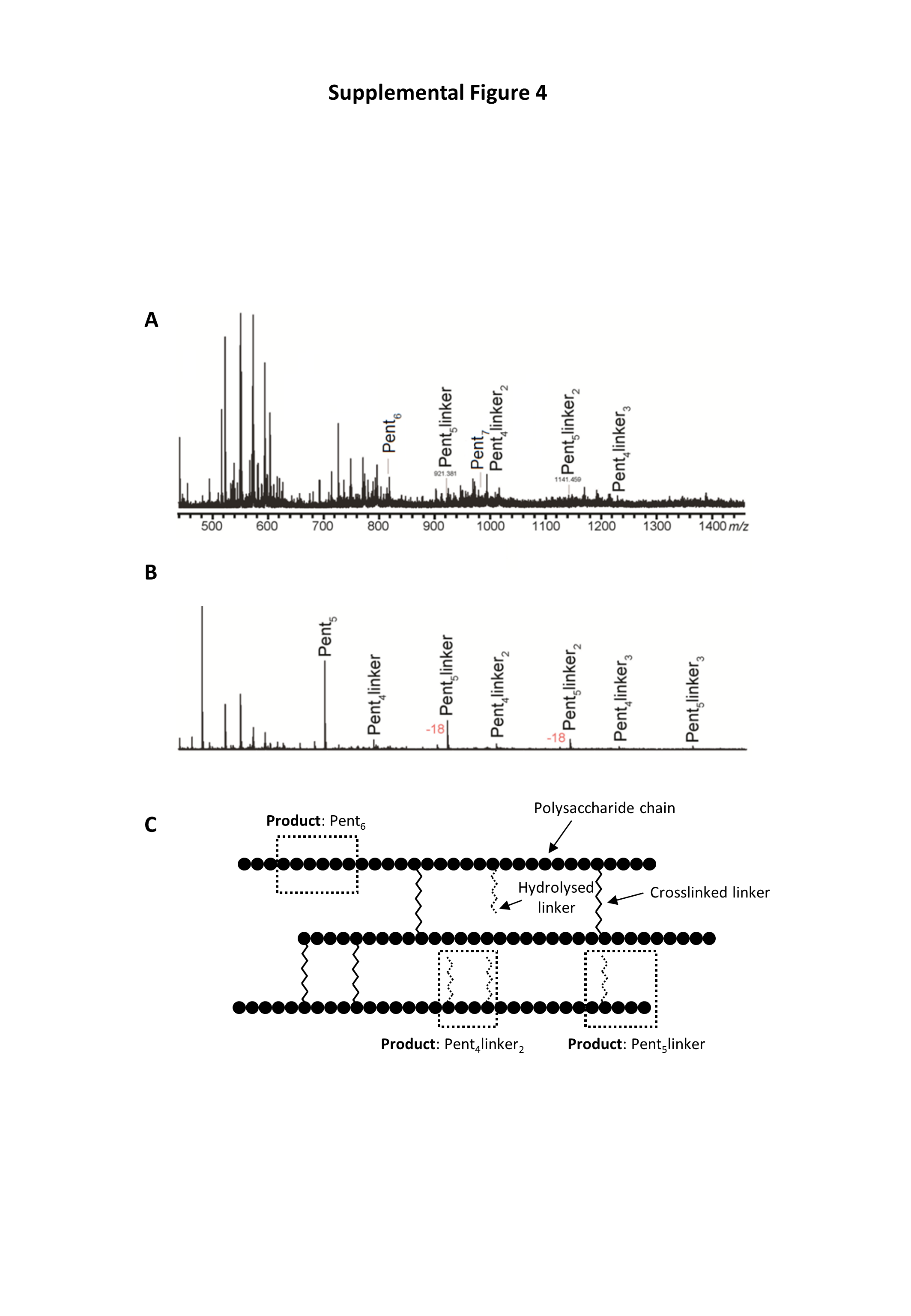

Supplement: Additional file 4: Figure S4. — Mass spectra of products from CPH-xylan digestion. (A) Mass spectrum of dyed CPH-xylan digested by xylanase xyl1 where the oligomers are composed of x pentose units and y hydrolysed linker units (Pentxlinkery). (B) Mass spectrum of undyed cross-linked xylan digested by xylanase xyl1 where the oligomers are composed of x pentose units and y hydrolysed linker units (Pentxlinkery). (C) Theoretical scheme showing the possible origins of the products observed in (A) and (B). [file 13068_2015_250_MOESM4_ESM.tif]

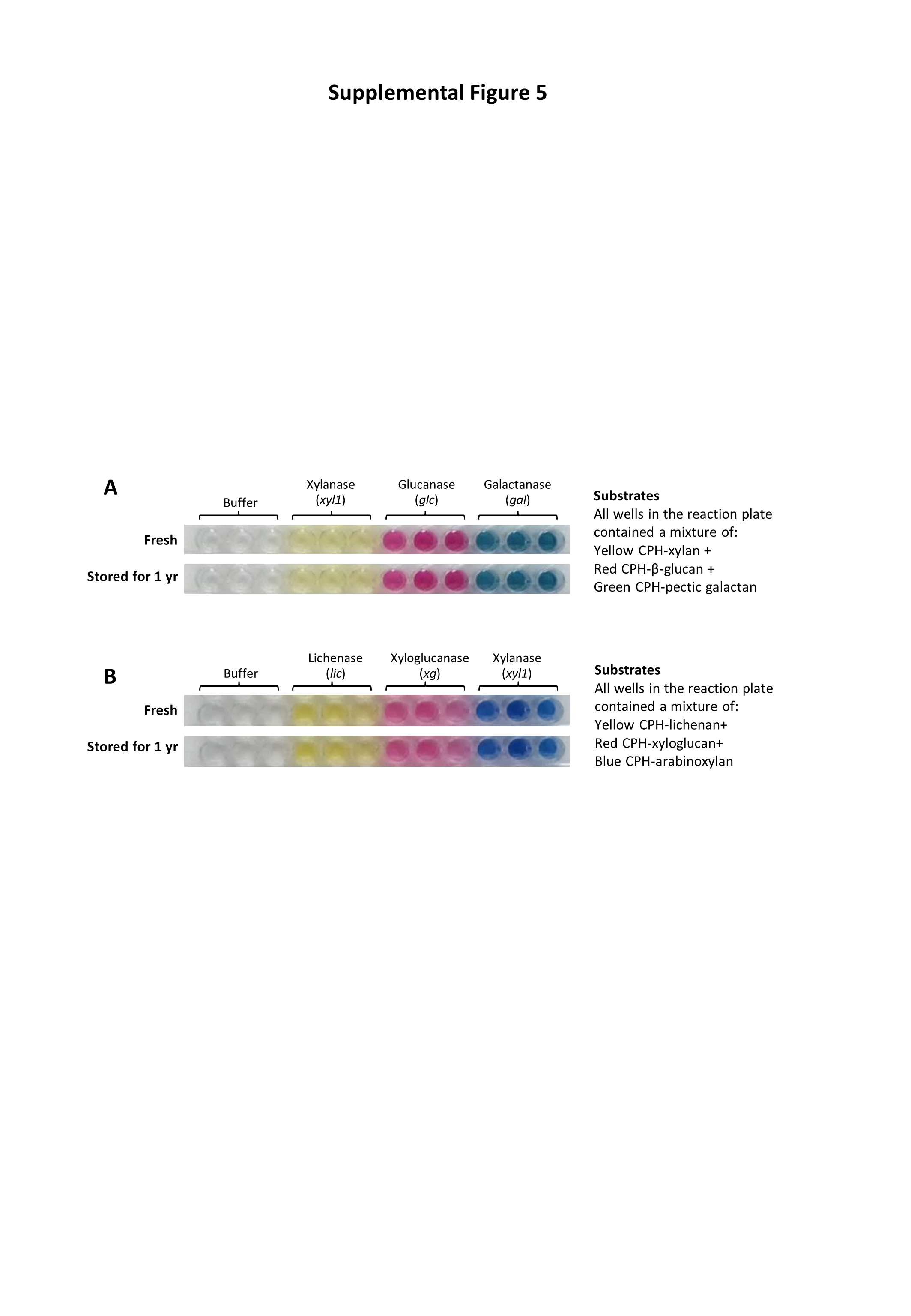

Supplement: Additional file 5: Figure S5. — Storage stability of CPH substrates. The storage stability of CPH substrates was tested by comparing the output from fresh and year-old reaction plates. (A) Images showing products from fresh and year-old reaction plates in which each well contained a mixture of the substrates shown to the right. The enzymes used are indicated at the top. (B) Another second example of an experiment comparing the output from fresh and year-old plates. Each well contained a mixture of the substrates shown to the right. The enzymes used are indicated at the top. See Tables 1 and 2 for details of the substrates and enzyme used. [file 13068_2015_250_MOESM5_ESM.tif]

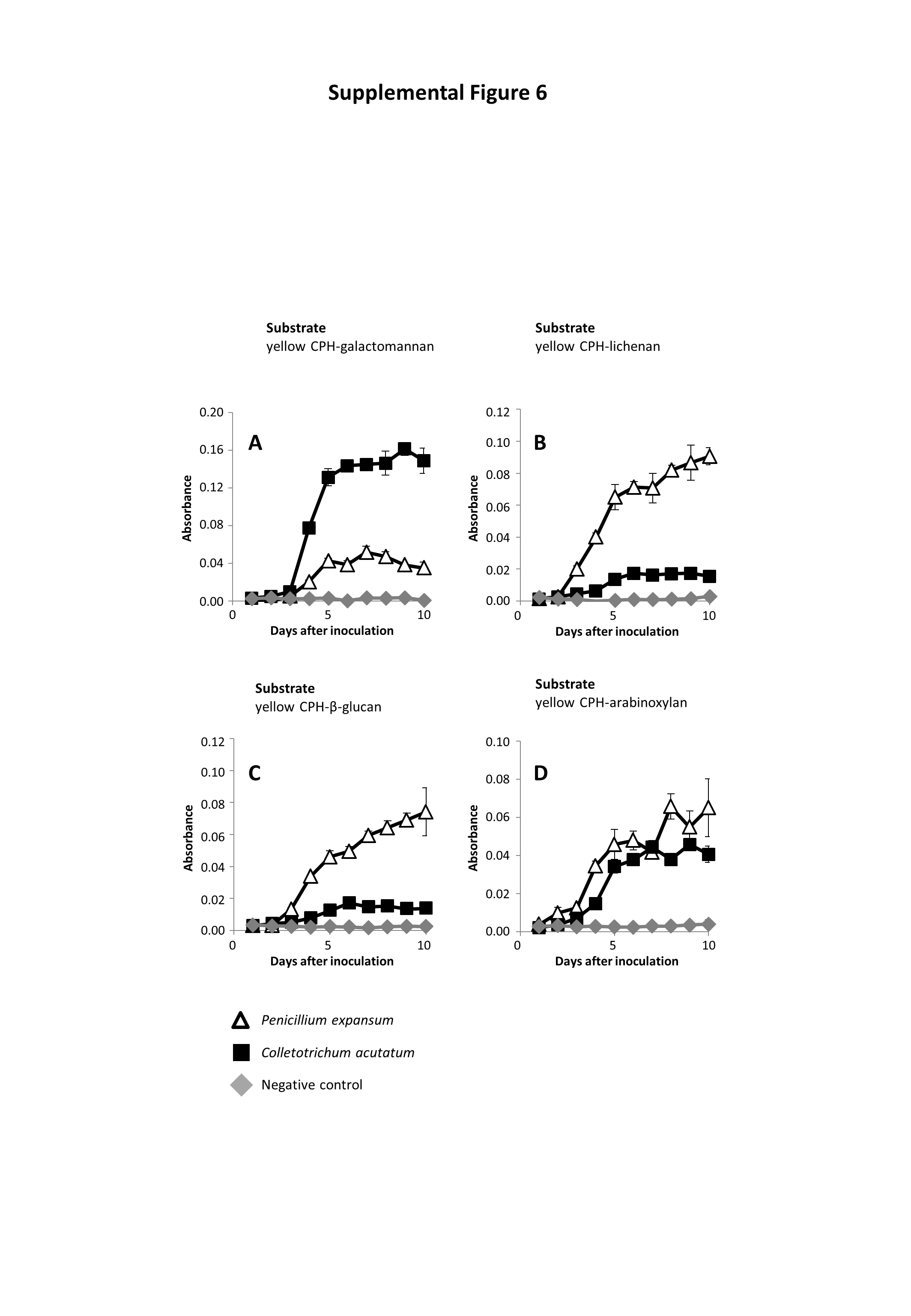

Supplement: Additional file 6: Figure S6. — Using CPH substrates to analyse enzyme activity in crude fungal broths. The two pathogenic fungi Penicillium expansum and Colletotrichum acutatum were cultivated over 10 days, and enzyme activities in the culture broth were analysed using four different CPH substrates: (A) yellow CPH-galactomannan, (B) yellow CPH-lichenan, (C) yellow CPH-β-glucan from barley, (D) yellow CPH-arabinoxylan. As a negative control, the substrates were also treated with broth alone (grey lines). The reaction was incubated in 100 mM sodium acetate buffer pH 4.5 for 1 h at room temperature. See Table 2 for details of the substrates used. [file 13068_2015_250_MOESM6_ESM.tif]

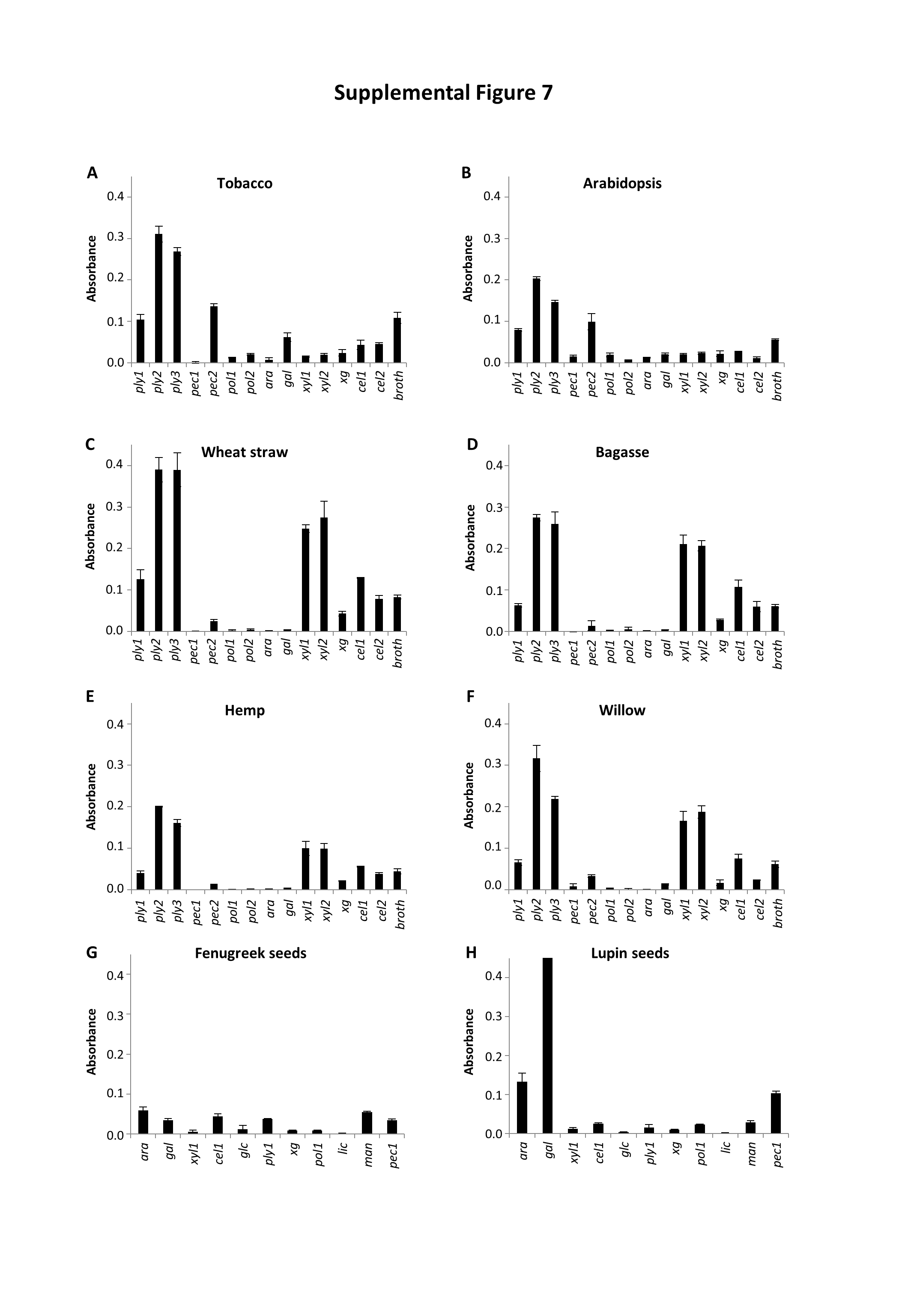

Supplement: Additional file 7: Figure S7. — Insoluble chromogenic biomass substrates. A range of insoluble chromogenic biomass substrates were produced (all using a red-coloured dye) and treated with a range of enzymes (shown on x-axes). The absorbance values are means from three wells measured at 517 nm. Enzymes were used at 10 U/mL for 24 h at room temperature. The assay was performed using 100 mM sodium acetate buffer pH 4.5, except for the enzymes ply1, ply2, ply3, pol1 and xg used at pH 5.5, for cel2 pH 6.0, for pec3 100 mM sodium phosphate pH 7.0 and for pec1 sodium carbonate pH 10.0. The substrates used were (A) tobacco, (B) Arabidopsis, (C) wheat straw, (D) bagasse, (E) hemp, (F) willow, (G) fenugreek seeds and (H) lupin seeds. See Table 2 for details of the enzyme used. [file 13068_2015_250_MOESM7_ESM.tif]

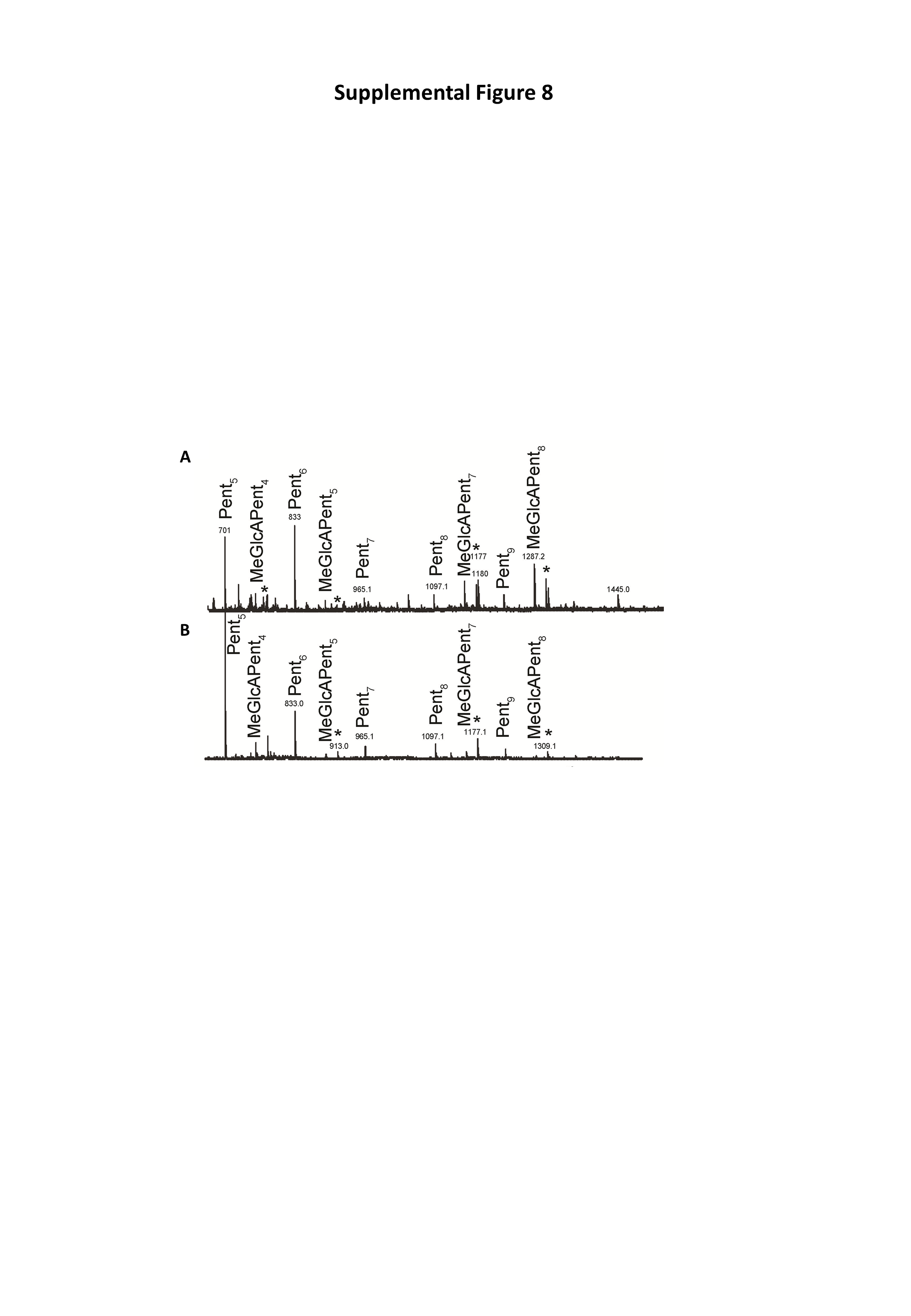

Supplement: Additional file 8: Figure S8. — MALDI-ToF MS spectra of released products during enzyme treatments of insoluble chromogenic biomass substrates. (A) MALDI-ToF MS spectra of the products released from hemp insoluble chromogenic biomass substrates treated with xylanase xyl1. (B) MALDI-ToF MS spectra of the products released from bagasse treated with xylanase nz2. MeGlcAxPenty represent oligosaccharides with x number of 4-methyl-glucuronic acid and y number of xylose residues; Pentx = pentose oligosaccharides (most likely (arabino-)xylooligosaccharides, arabinosylations limiting complete degradation by the endoxylanases xyl1 and nz2) containing x number of pentoses; asterisk (*) represents sodium adducts, which commonly occur for uronic acids. [file 13068_2015_250_MOESM8_ESM.tif]
